# Supplementary material for: Lignin as a High-Value Bioaditive in 3D-DLP Printable Acrylic Resins and Polyaniline Conductive Composite
Source: Polymers (Basel). 2022 Oct 4;14(19):4164. doi: 10.3390/polym14194164 (PMC9572831; doi:10.3390/polym14194164)
Supplement: Supplementary file 1 [file polymers-14-04164-s001.zip › polymers-1945893-supplementary.pdf]

Supporting Information

Lignin as a High-Value Bioaditive in 3D-DLP Printable Acrylic Resins and Polyaniline Conductive Composite

Goretti Arias-Ferreiro[1], Aurora Lasagabáster-Latorre[2], Ana Ares-Pernas[1], Pablo Ligero[3], Sandra María García-Garabal[4], M. Sonia Dopico-García[1], María-José Abad[1]

[1] Universidade da Coruña, Campus Industrial de Ferrol, Grupo de Polímeros-CITENI, Campus de Esteiro, 15403 Ferrol, Spain

[2] Dpto Química Orgánica I, Facultad de Óptica, Universidad Complutense de Madrid, Arcos de Jalón 118, 28037 Madrid, Spain

[3] Enxeñería Química Ambiental Group, Centro de Investigacións Científicas Avanzadas (CICA), Universidade da Coruña, 15071, A Coruña, Spain

[4] Grupo Mesturas. Universidade da Coruña, Campus da Zapateira s/n 15071 A Coruña, Spain

*Corresponding author **maria.jose.abad@udc.es**

S1. RESULTS

S1.1. RHEOLOGY

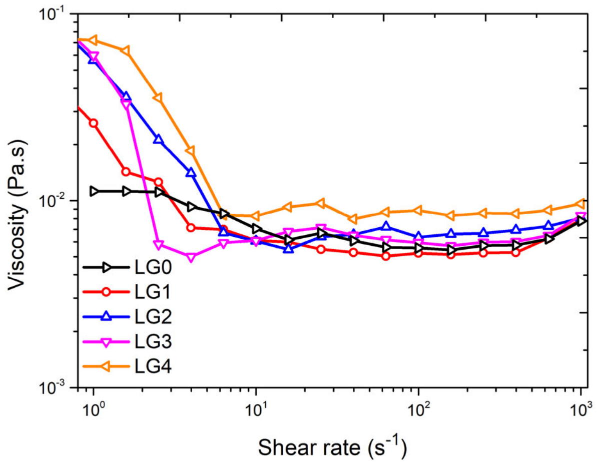

Figure S1. *Viscosity values of liquid formulations as a function of shear rate and Lignin amount at room temperature*

S1.2. MORPHOLOGY OF THE FILLERS.

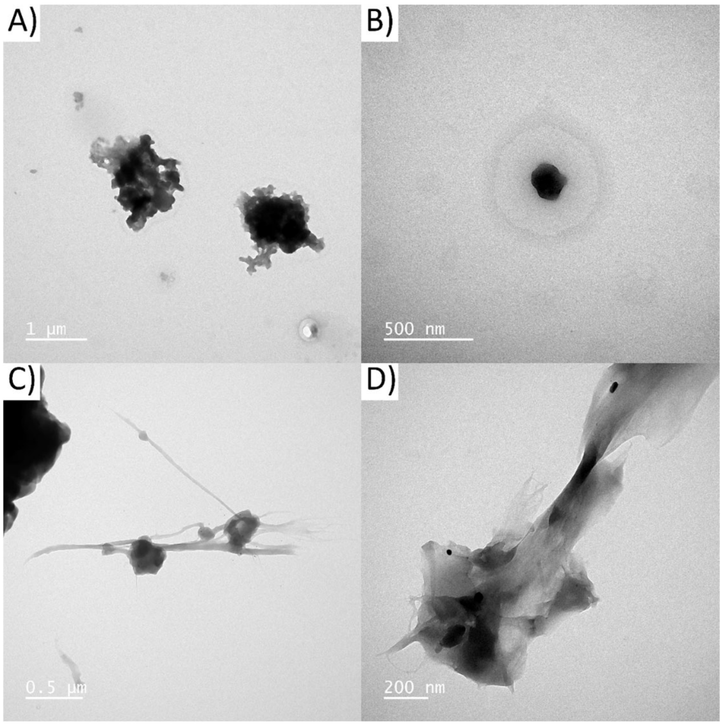

Figure S2. *TEM images of A and B) PANI and C and D) Lignin*

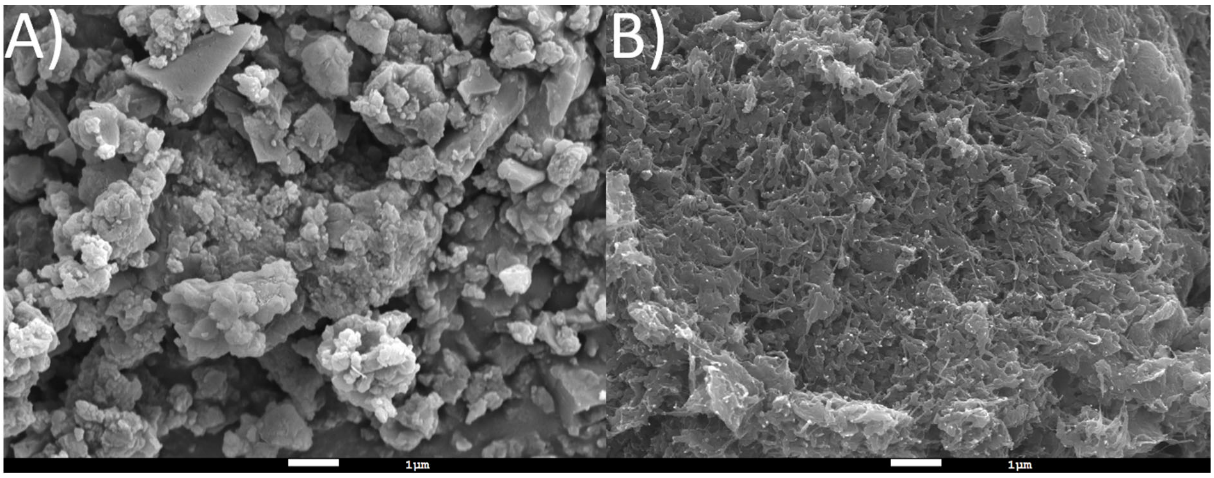

Figure S3. *Representative SEM images of A) PANI and B) Lignin with magnitude amplification of 10000x*

S1.3. FTIR spectroscopy of pTSA-PANI

The KBr FTIR spectrum of the synthesized p-TSA-PANI is plotted in **Figure S6**. All the characteristic peaks of PANI plus those of the dopant, p-Toluensulphonic acid, are perceived demonstrating that PANI is in the protonated doped state. The intense broad peak at 3425 cm^{-1} is due to overlapping of NH stretching from PANI and OH stretching (ν_{OH}) from the $–SO_3H$ and their interactions. The main PANI bands are detected at 1561 cm^{-1} (Quinoid rings, Q), 1467 cm^{-1} (Benzenoid rings, B), 1301 cm^{-1} ($\nu_{C–N}$), 1223 cm^{-1} ($\nu_{C–N+}$ in the polaron structure), 1119 cm^{-1} (combination of δ_{C-H}, $\delta_{Q-N+-B/B-NH.-B}$, considered a measure of the degree of electron delocalization and is characteristic of PANI conductivity) and 800 cm^{-1} (aromatic C-H out-of-plane bending vibration on a 1,4-disubstituted aromatic rings, γ_{C-H})[1].

The absorption peaks at 1064 and 1009 (asymmetric and symmetric $\nu_{O=S=O}$), and 621 cm^{-1} (ν_{C-S}) in the FTIR spectrum are consistent with the existence of the $-SO_3-$ group attached to the aromatic rings[2]. Finally, the band centered at 880 cm^{-1} indicates the presence of HSO_4- or $SO4^{-2}$ counterions associated with the use of APS as oxidant[3].

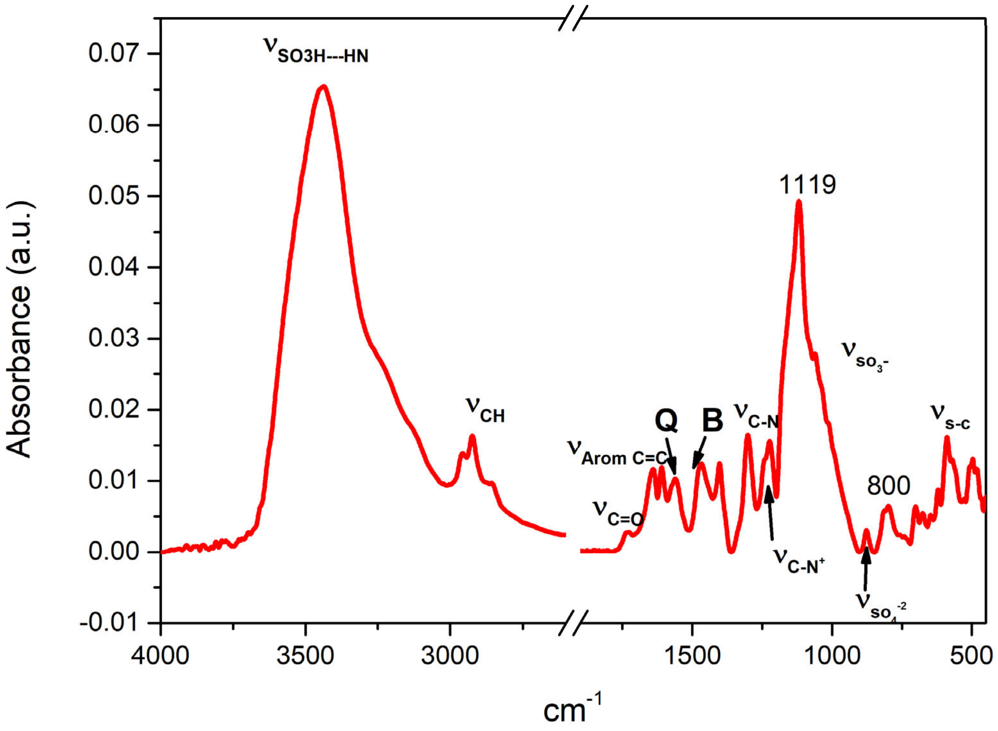

Figure S4. *FTIR spectrum of p-TSA-PANI*

S1.8. MORPHOLOGY OF p-TSA-PANI PRINTED COMPOSITES

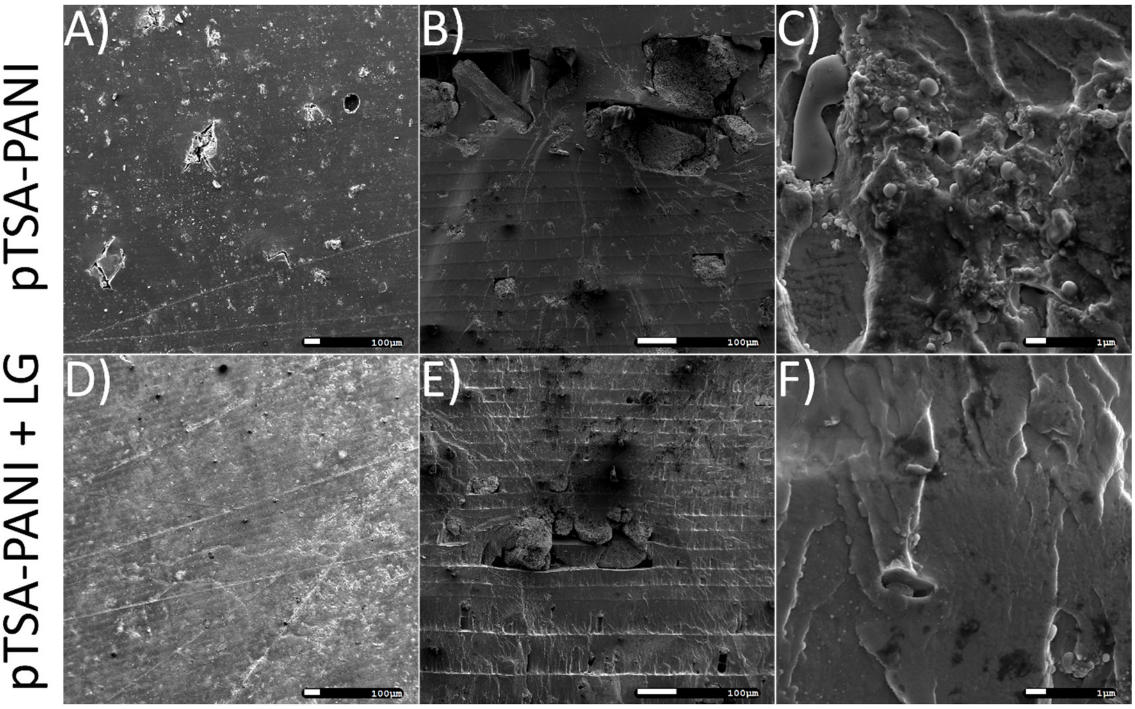

Figure S5. *Representative SEM images of (A) LG0PANI5 surface with magnitude amplification of 40×, (B) LG0PANI5 cryo-fractured cross-sections with magnitude amplification of 100x and C) X5000. (D) LG1PANI5 surface with magnitude amplification of 40×, (E) LG1PANI5 cryo-fractured cross-sections with magnitude amplification of 100x and F) X5000*

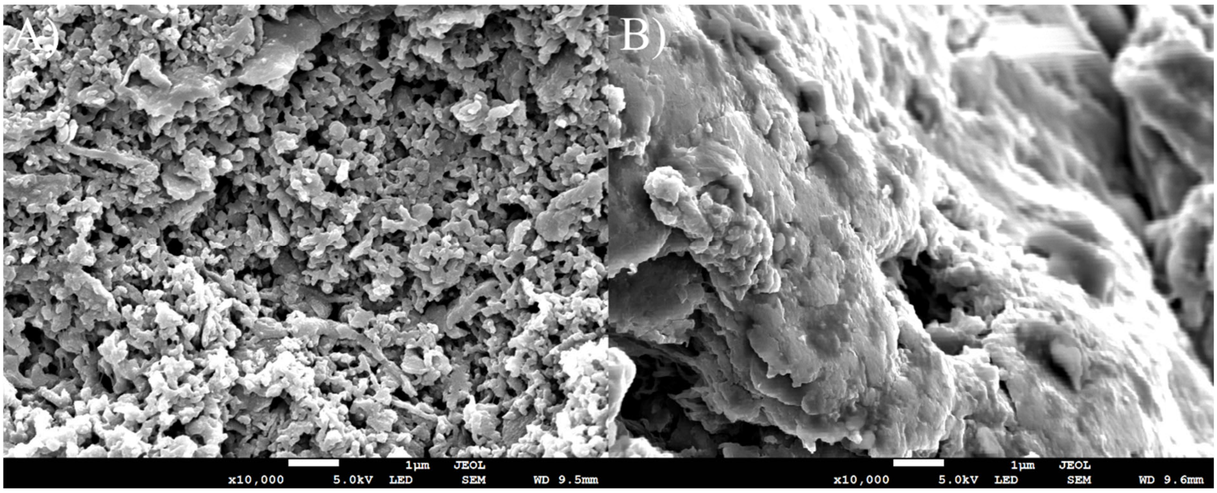

Figure S6. *SEM images of cryo-fractured cross-sections with magnitude amplification of 10000x (A) LG0PANI5 and B) LG1PANI5*

REFERENCES

[1] F. Usman, J. O. Dennis, A. Y. Ahmed, K. C. Seong, Y. W. Fen, A. R. Sadrolhosseini, F. Meriaudeau, P. Kumar, O. B. Ayodele, *J. Mater. Res. Technol.* **2020**, *9*, 1468.

[2] M. Khalid, M. A. Tumelero, I. S. Brandt, V. C. Zoldan, J. J. S. Acuña, A. A. Pasa, *Indian J. Mater. Sci.* **2013**, *2013*, 1.

[3] L. Horta Romarís, M. V. González Rodríguez, B. Huang, P. Costa, A. Lasagabáster Latorre, S. Lanceros-Mendez, M. J. Abad López, *J. Mater. Chem. C* **2018**, *6*, 8502.
